# An Evolutionary Field Theorem: Evolutionary Field Optimization in Training of Power-Weighted Multiplicative Neurons for Nitrogen Oxides-Sensitive Electronic Nose Applications

**DOI:** 10.3390/s22103836

**Published:** 2022-05-18

**Authors:** Baris Baykant Alagoz, Ozlem Imik Simsek, Davut Ari, Aleksei Tepljakov, Eduard Petlenkov, Hossein Alimohammadi

**Affiliations:** 1 Department of Computer Engineering, Inonu University, Malatya 44000, Turkey; oimiksimsek@gmail.com; 2 Department of Computer Engineering, Bitlis Eren University, Bitlis 13000, Turkey; dari@beu.edu.tr; 3 Department of Computer Systems, Tallinn University of Technology, 12618 Tallinn, Estonia; aleksei.tepljakov@taltech.ee (A.T.); eduard.petlenkov@taltech.ee (E.P.); hossein.alimohammadi@taltech.ee (H.A.)

**Keywords:** neuroevolution, evolutionary optimization, multiplicative neuron model, concentration estimation, electronic nose, Industry 4.0

## Abstract

Neuroevolutionary machine learning is an emerging topic in the evolutionary computation field and enables practical modeling solutions for data-driven engineering applications. Contributions of this study to the neuroevolutionary machine learning area are twofold: firstly, this study presents an evolutionary field theorem of search agents and suggests an algorithm for Evolutionary Field Optimization with Geometric Strategies (EFO-GS) on the basis of the evolutionary field theorem. The proposed EFO-GS algorithm benefits from a field-adapted differential crossover mechanism, a field-aware metamutation process to improve the evolutionary search quality. Secondly, the multiplicative neuron model is modified to develop Power-Weighted Multiplicative (PWM) neural models. The modified PWM neuron model involves the power-weighted multiplicative units similar to dendritic branches of biological neurons, and this neuron model can better represent polynomial nonlinearity and they can operate in the real-valued neuron mode, complex-valued neuron mode, and the mixed-mode. In this study, the EFO-GS algorithm is used for the training of the PWM neuron models to perform an efficient neuroevolutionary computation. Authors implement the proposed PWM neural processing with the EFO-GS in an electronic nose application to accurately estimate Nitrogen Oxides (NO_x_) pollutant concentrations from low-cost multi-sensor array measurements and demonstrate improvements in estimation performance.

## 1. Introduction

Evolutionary neural networks have been in progress, and the neuroevolution, which enables cooperation of evolutionary computation with neural information processing, contributes to improvements of Artificial Neural Network (ANN) models in data-driven real-world applications [1,2,3,4,5,6,7]. Evolutionary optimization has been used for both architecture optimization of neural network models [8,9] and training of neural networks [5]. However, data-driven evolutionary optimization was shown to be effective in solving real-world problems [10,11]. A comprehensive review of data-driven evolutionary optimization and its engineering applications have been presented in [11]. A prominent advantage of the evolutionary optimization method comes from the easy employment of genetic and evolutionary search processes in searching solutions of very sophisticated optimization problems [11], and this property can facilitate the training process of neural networks in cases that the gradient-based optimization method is not feasible to apply; particularly when basic elements of neural models involve very complicated mathematical statements or gradient calculations are not valid. In addition, multi-agent search of population-based metaheuristic methods allows finding more optimal training solutions compared to single-agent search methods that can perform a local search. The search performance of single-agent search methods is very dependent on the initial conditions and configurations.

There are several works that have reported improvements of ANN training performance by means of population-based metaheuristic optimization methods: Sexton et al. revealed that a genetic algorithm could be preferred for the training task of shallow neural networks, and they reported a superior training performance of a genetic algorithm over the backpropagation methods [12]. In a similar work, Che et al. concluded that the backpropagation algorithm could provide faster training of ANNs than the genetic algorithm (GA); however, it could suffer from a gradient-vanishing problem whereas the genetic algorithm does not suffer [13]. Swarm-based search algorithms were also implemented in ANN training process: Gudise et al. compared the neural network training performance of particle swarm optimization (PSO) with performance of a backpropagation algorithm. They claimed that the particle swarm optimization algorithm could converge faster to optimal weights than the backpropagation algorithm [14]. Table 1 briefly summarizes some advantages and disadvantages of fundamental metaheuristic optimization methods for the training of ANNs. In the literature, several contemporary metaheuristic algorithms were compared for the training of neural networks [15,16,17,18]. Besides the weight optimization for the training of ANN, evolutionary optimization methods have also been preferred for the optimization of neural network architectures [8,9]. The differential evolution (DE) algorithms are also very effective evolutionary search algorithms [19,20,21,22] and they were used for training of several types of neural networks [23,24] and configuration of neural network parameters [25,26]. Through these progresses, neuroevolutionary computation has become a popular topic that involves evolutionary training and architectural optimization of neural networks [6].

The origin of evolutionary computation has strong connection with evolutionary biology and evolution theory. Algorithms of evolutionary computation may be inspired by evolution mechanisms of individuals and species in macroscopic and microscopic scales and genetics of organisms at the molecular biology level [29,30,31]. In the current study, we proposed an evolutionary optimization method on the basis of an Evolutionary Field Theory (EFT) of search agents. To the best of our knowledge, the term of evolutionary field theory was used by Papadopoulos et al. and they established the stochastic field model of uncertain systems by using non-homogeneous evolutionary fields developed by Priestley [32,33]. These works considered this term for spectral analysis of non-stationary processes according to the concept of evolutionary spectra, where spectral functions were assumed to be time-dependent [34]. In the current study, the term of evolutionary field is used for a property space where search agent properties evolve in time, to better fit to the solution environment of a predefined optimization problem. Therefore, we conjecture an evolutionary field theory of the search agents in order to establish a theoretical foundation for analysis and design of population-based evolutionary algorithms from an agent–environment perspective that is very similar to the basics of the reinforcement learning [35]. This theorem can establish a bridge between majority of population-based evolutionary search algorithms and the reinforcement learning foundation collection. The contributions of this study are twofold:

(i) Suggestion of an evolutionary field optimization;
(ii) Development of a PWM neural processor for evolutionary nonlinear programming in data-driven applications.

For the training of this PWM neural processor, we suggest an evolutionary metaheuristic optimization method. The proposed algorithm is referred to as Evolutionary Field Optimization (EFO) because it is based on the evolutionary field theory of search agents. This algorithm was suggested to facilitate the training of the power-weighted multiplicative neuron models by implementing neuroevolutionary machine learning in this study. The EFO algorithm performs field-adapted geometric search strategies in the evolution field that was composed of property codes of search agents. The proposed EFO-GS algorithm implements a hybrid search strategy that combines advantages of a geometric space search strategy with differential evolutionary search mechanisms. Then, the EFO-GS is implemented for the optimization of weight parameters in training of PWM neuron models in this study. We provide a deepened analysis on features of the multiplicative neuron models, and suggest a power-weighted multiplicative neuron model in order to manage three different operation modes of this neuron, which are the real-valued neuron mode, the complex-valued neuron mode, and the mixed-mode operations. To address the solution of real-valued regression problems, the multiplicative neuron model is modified by appending a special type activation function, which is referred to as the mapping-to-real function. This function maps dual properties of complex numbers (real-imaginary parts or magnitude-phase properties) to a real value. Thus, this extension enables us to convert results in the complex-valued domain of the neuron into a real-valued signal at the neuron output. A practical application of a PWM neuron model with EFO training (PWM-EFO) was demonstrated for estimation of NO_x_ concentration to achieve accurately the soft-calibration of a low-cost multisensor array. An experimental study was conducted and the effectiveness of the proposed estimation models was demonstrated for electronic nose applications.

### A Brief Review of Pathways from Additive Neurons and Multiplicative Neurons

In order to perform practical machine learning tasks, ANNs have been widely preferred for the identification of black-box models from very sophisticated and noisy data stacks. Originally, the topic of ANN model can be traced back to the suggestion of a simple artificial neural cell model by the physiologist Warren McCulloch and the mathematician Walter Pitts in 1943 [36]. However, harnessing the learning power of ANNs has begun after Rosen Blatt’s multilayer perception and proposition of the backpropagation algorithm [37]. These progresses have been milestones on the headway of deep neural networks. Then, a variety of application areas have emerged, such as in modeling [38], control [39], signal processing [40], image processing [41]. The backpropagation algorithm has been widely preferred training algorithm for multilayer feedforward ANNs in order to establish a neural model of the input–output relations in the training datasets [42]. Although there exists several variants of backpropagation algorithms, the Levenberq–Marquardt (LM) algorithm is widely used since it provides an enhanced training performance for feedforward multilayer neural networks [43,44]. The role of activation functions and design of parametric activation functions by using evolutionary methods were discussed in [45].

An extension of basic neuron model, known as multiplicative neural networks, emerged in the 1980s. Use of multiplicative units in neurons and their high-order model representation capability were discussed by Giles et al. [46]. Later, Durbin and Rumelhart suggested a multiplicative neural network structure. Thus, product units have been considered as a new form of computational unit for feedforward neural networks. They conjectured that the multiplication unit was more biologically plausible and more computationally powerful than the addition unit [47]. Another work reported that the multiplicative neural network can solve some problems by using less neurons than additive neurons and heuristic methods were suggested for the solution of training problems of multiplicative networks [48]. Afterwards, Schmitt investigated the computation complexity and learning skills of multiplicative neural networks and provided a detailed survey of their biological and computational origins [49]. Besides its computational origin, multiplicative neural activity has some neurobiological bases: from a neurobiological standpoint, Salinas and Abbott reported that multiplicative neural responses could arise in the overall responses of the neuron population in parietal cortex, and the multiplicative gain modulation could play an important role in the transformation of object locations from the retinal to body-centered coordinates. They conjectured that neurons with multiplicative responses can act as powerful computational elements in biological neural networks [50]. Main reason for this analogy with multiplication is that nonlinear relations in a neural system can be better represented by the product units than the additive units in modeling. In the machine learning domain, Simon revealed an interesting correspondence between the multiplicative neuron and the additive neuron, according to the identify $\prod_{j}^{}x^{p_{i,j}}=e^{\sum_{j}^{}p_{i,j}In\left(x_{j}\right)}$ [47], and remarked that the multiplicative neuron network can be expressed in the form of an additive neuron network with a different nonlinearity [51]. At another venue, polynomial neural networks have been in progress, and their advantage over additive networks has been investigated [52,53,54]. Relations between polynomial regression and classical neural networks were discussed and polynomial activation functions were shown on the basis of the Taylor theorem [54].

## 2. Evolutionary Field Search

In general, population-based evolutionary algorithms (e.g., genetic algorithm, differential evolution, particle swarm optimization, etc.) implement a collection of search agents that iteratively repositions the solution in the search space of an optimization problem, to find a better solution point during the optimization process. Repositioning of search agents is commonly performed by predefined evolution rules; for instance, fundamental genetic processes for genetic algorithm, motion equations for swarm-based metaheuristic optimization methods. On the other hand, the field of reinforcement learning is closely related with optimization of agent response in an environment from its experience [35]. This study provides a bird’s eye view for the population-based evolutionary search algorithms from the perspective of the reinforcement learning. This theorem may be also useful for analysis and design memetic algorithms and memetic computing [55]. The following section aims to present an evolutionary field theorem that has a common aspect or establishes a common foundation in the analysis of these type search agents.

### 2.1. Evolutionary Field Theorem of Search Agents

**Figure 1:**
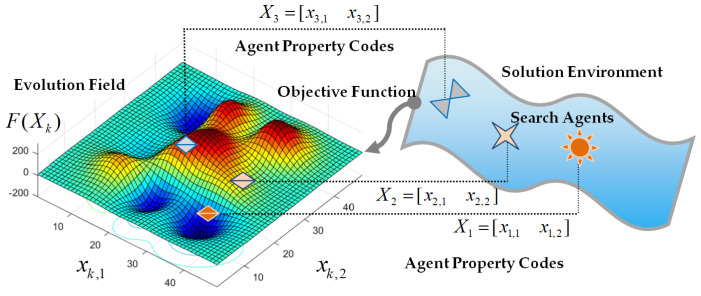
A schematic view of property codes in the evolution field and associated agents in solution space of the optimization problem.

The evolution field is a multi-dimensional space of agent property code, where agent properties are represented by a property code $X_{k}\in R^{D}$ (the parameter *D* is the dimension of the property code and *k* is the agent index) and evolve in time. Search agents, which are characterized by their property code, only act in a solution environment of optimization problems, and each agent represents an individual in the solution environment. Commonly, a selection mechanism is designated such that their chances of survival depend on the fitness of the agent to the solution environment. Therefore, the objective function $F(X_{k})$ measures the suitability of agent property code to represent an optimal solution of the environment, and the value of $F(X_{k})$ expresses the field value of the property code, and the field value has been widely used in the reposition of agent property codes within the evolution field. Hence, the field value is considered for the evolution of agents and selection of them. In essence, objectives assign a suitability value to the property code of the search agent in the evolutionary field. The evolutionary field can be defined by a closed set of property codes and the objective function in the form ($X_{k},\;F\left(X_{k}\right)$), and this closed set is a minimal set to design evolution strategies of agent properties in the field. Figure 1 depicts the evolution field of the property codes and their association with the agents of the solution environment. The property code is represented by a vector $X_{k}$, where vector elements $x_{i,j}$ represent the *j*th property of the search agent *k*.

(1) $$X_{k}=\left[\begin{array}{cccc} x_{k,1} & x_{k,2} & \ldots & x_{k,D} \end{array}\right]$$

To manage geometrical evolution strategies of property codes, agent properties are widely embedded into a Cartesian coordinate system. Thus, distance metrics become valid in order to express evolutionary relations between the agent property codes. Consequently, property codes in Cartesian coordinates establish a metric space ($X_{k},d$) where the operator *d* represents a metric that holds:

1. $d(X_{i},X_{j})\geq 0$—the metric $d(X_{i},X_{j})$ expresses dissimilarity of agent properties in the defined metric space. The equality state $d(X_{i},X_{j})=0$ implies that the agent *i* and the agent *j* are the same agent in the solution environment. Values of $d(X_{i},X_{j})$ can express a measure for differentiation of agent properties, and it can be used to evaluate the amount of the evolution of the agent property code;
2. $d\left(X_{i},X_{j}\right)=d\left(X_{j},X_{i}\right)$—agent properties do not apply any priority;
3. $d\left(X_{i},X_{j}\right)\leq d\left(X_{i},X_{k}\right)+d\left(X_{k},X_{j}\right)$—agent properties obey the triangle inequality and it allows to define geometrical evolution strategies. The shortest evolutionary path does not involve any deflection in the code space.

Relocation of the agent property code in the evolution field results in the change of agent properties, and the property code relocation is referred to as evolution of the agent. Amount of evolution can be expressed by the distance metric in the evolution field. Let us assume the property code ($X_{k}\left[n\right]$) of the agent *k* at the instance *n* changes to a property code $X_{k}\left[n+1\right]$ at the instance n+1. The amount of evolution can be measured at this instance by the seasonal evolution that satisfies

(2) $$d(X_{k}\left[n\right],X_{k}\left[n+1\right])\geq 0.$$

The seasonal evolution rate in the property code of agent *k* can be written as

(3) $$E_{r}\left(X_{k}\left[n\right]\right)=\frac{d(X_{k}\left[n\right],X_{k}\left[n+1\right])}{∥X_{k}\left[n\right]∥}.$$

where the operator $∥X_{k}\left[n\right]∥$ represents the norm of $X_{k}\left[n\right]$ vector. Evolution of some agent properties may be advantageous for survival of agents that act in the solution environment, and some may not be advantageous. The higher field value $F(X_{k}\left[n\right])$ infers a higher tendency to evolve, and the evolutionary energy density at the code $X_{k}\left[n\right]$ can be expressed by the value of $F(X_{k}\left[n\right])$. The advantageous evolution can be perceived with the condition $F\left(X_{k}\left[n+1\right]\right)<F\left(X_{k}\left[n\right]\right)$. Then, the negative derivative condition to minimize the field value (evolutionary energy density) of agent *k* can be written on the basis of the Lyapunov stability as

(4) $$\Delta F_{k}\left[n\right]=F\left(X_{k}\left[n+1\right]\right)-F\left(X_{k}\left[n\right]\right)<0.$$

(See Remark A1 in Appendix A for the mathematical foundation of this negative derivative condition.) Useless seasonal evolution can be detected by checking the condition $F\left(X_{k}\left[n+1\right]\right)\geq F\left(X_{k}\left[n\right]\right)$. Since the evolution is a continuing process, a useful evolutionary path for agent property *k* can be expressed for *L* number of seasons as follows:

(5) $$\Delta E_{k}\left[L\right]=\sum_{n=i}^{i+L}\Delta F_{k}\left[n\right]<0$$

To our knowledge, selection mechanisms in nature may not always have to know or be aware of the most advantageous path in a long horizon of the evolution process. Therefore, the selection mechanism in nature can be assumed to behave in the manner of Markovian processes; useful transitions of agent properties, namely seasonal advantageous evolution of the property code can be expressed according to the current state of field values as

(6) $$X_{k}\left[n+1\right]=\left\{\begin{array}{cc} X_{k}\left[n+1\right] & F\left(X_{k}\left[n+1\right]\right)-F\left(X_{k}\left[n\right]\right)<0\\ X_{k}\left[n\right] & F\left(X_{k}\left[n+1\right]\right)-F\left(X_{k}\left[n\right]\right)\geq 0 \end{array}\right\}.$$

**Figure 2:**
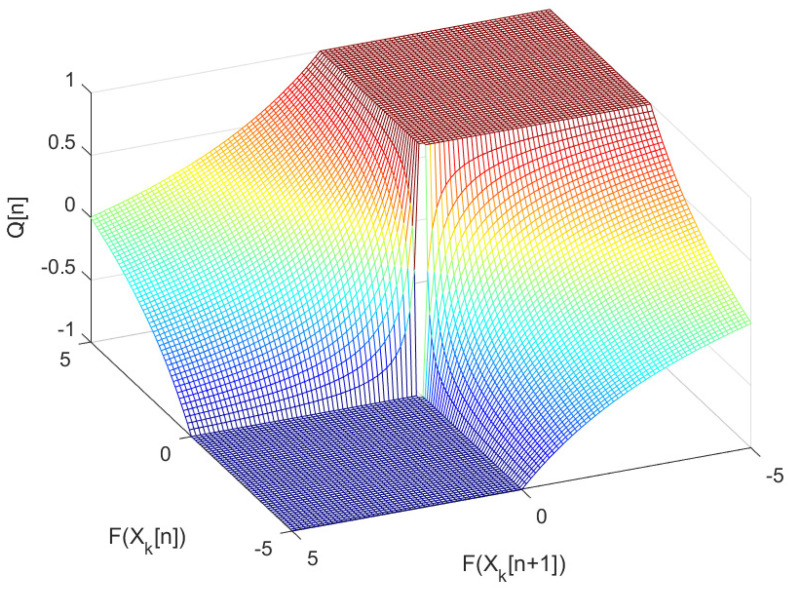
Values of seasonal quality index Q[n] according to values of $F(x_{k}\left[n+1\right])$ and $F(x_{k}\left[n\right])$.

Quality of the property evolution can be expressed by the loss in evolutionary energy, and it can be written as

(7) $$Q\left[n\right]=-\frac{F\left(X_{k}\left[n+1\right]\right)-F\left(X_{k}\left[n\right]\right)}{\left|F(X_{k}\left[n+1\right])\right|+\left|F(X_{k}\left[n\right])\right|},$$

where the seasonal quality index Q[n] takes a value [−1, 1]. A value of −1 implies the low quality, and the value of +1 implies the high quality in seasonal evolution. Figure 2 illustrates values of the seasonal quality index Q[n] for the sampled values of $F(X_{k}\left[n+1\right])$ and $F(X_{k}\left[n\right])$ in a value set of [−5, 5].

### 2.2. An Evolutionary Field Optimization with Geometric Strategies

**Figure 3:**
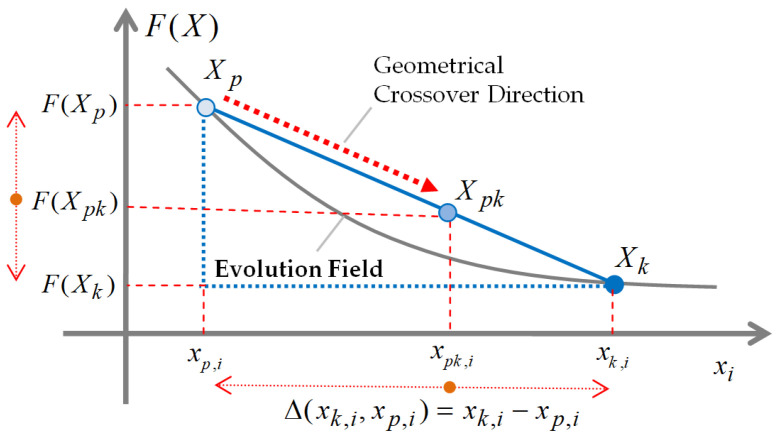
An illustration that describes the field-adapted differential crossover between $x_{k,i}$ and $x_{p,j}$ components of property codes $X_{k}$ and $X_{p}$.

EFO-GS algorithm implements a hybrid search methodology that aims to benefit from advantages of the geometrical search strategies in the evolutionary field to improve the differential evolution processes. Effective geometrical search methods have been shown to convergence to minimum points [56,57,58]. The EFO-GS algorithm evolves an initial property code $X_{k}$ towards the seasonal best of codes ($X_{best}\left[n\right]=\underset{X_{j}}{argmin}F\left(X_{j}\left[n\right]\right)$, $j=1,2,3,\ldots ,h_{k}$, the parameter $h_{k}$ is the number of agent population) by repeatedly performing advantageous seasonal evolution of the property code according to scattering geometry of agent’s property codes within the evolution field. Two essential genetic mechanisms are employed to perform the seasonal evolution of agent properties:

(i) *Field-adapted differential crossover of property codes:* The property difference is expressed as

(8) $$\Delta \left(x_{k,i},x_{p,i}\right)=x_{k,i}-x_{p,i},$$

where $x_{k,i}$ is *i*th property of the property code $X_{k}$ and $x_{p,i}$ is *i*th property of the property code $X_{p}$. (To make the formulation simple and clear, we prefer to express element-wise formulations instead of vector or matrix forms.) The field-adapted differential crossover is performed by using the property difference $\Delta (x_{k,i},x_{p,i})$ according to a predefined geometrical rule on the field as depicted in Figure 3. Figure 3 depicts a geometrical interpretation for the field-adapted differential crossover for $x_{k,i}$ and $x_{p,i}$ components of property codes $X_{k}$ and $X_{p}$. To perform a high-quality property evolution in the convex part of the field, this geometrical rule was proposed as: each property in the property code performs a differential crossover with a magnitude of the seasonal quality factor $\left|Q[n]\right|$ towards the agent $X_{k}\left[n\right]$ with the lower field value $F(X_{k}\left[n\right])$. The $X_{pk,j}$ represents the evolved property of this geometric crossover rule. Arithmetically, this rule can be expressed as

(9) $$X_{pk,j}=x_{p,i}+\left(x_{k,i}-x_{p,i}\right)\left|Q[n]\right|,$$

(10) $$\left|Q[n]\right|=\frac{\left|F\left(X_{k}\left[n\right]\right)-F\left(X_{p}\left[n\right]\right)\right|}{\left|F(X_{k}\left[n\right])\right|+\left|F(X_{p}\left[n\right])\right|}.$$

**Figure 4:**
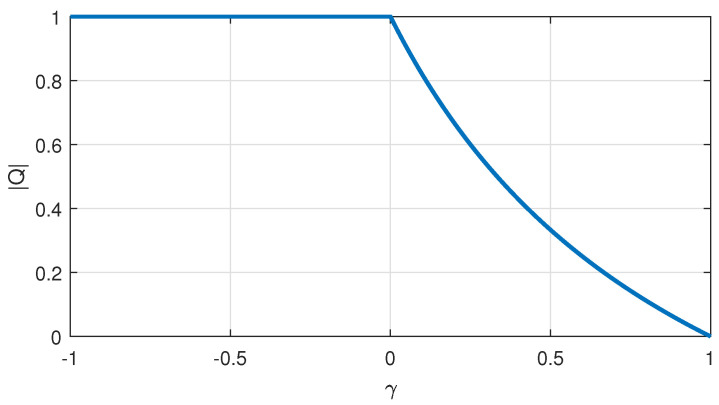
Change in the magnitude of seasonal quality factor $\left|Q[n]\right|$ for $F\left(X_{k}\right)=\gamma F\left(X_{p}\right)$.

It is useful to consider the change of quality factor magnitude depending on property codes. Figure 4 shows the magnitude of the seasonal quality factor for a relative change of the field values of the agent $X_{k}$ as $F\left(X_{k}\right)=\gamma F\left(X_{p}\right)$, γ∈[−1,1]. For γ=1, it implies $F\left(X_{k}\right)=F\left(X_{p}\right)$, and it leads to a zero value for the seasonal quality factor magnitude ($\left|Q[n]\right|$). No geometrical crossover is applied, and it yields $x_{pk,i}=x_{p,i}$ in this case. For γ≤0, it implies $F\left(X_{k}\right)\ll F\left(X_{p}\right)$, and it yields the highest value for the seasonal quality factor, meanwhile the geometrical differential crossover is fully performed toward to the property code $X_{k}$ that has the lower field value. Recently, a different definition of evolution quality and distance metrics were employed to improve differential evolution performance [21]; however, the algorithmic structure and evolution formulations in [21] are not the same as those of the EFO algorithm.

A property code search in locations around the seasonal best agent code $X_{best}\left[n\right]$ improves the convergence speed of the optimization process. This leads to a seasonal exploitation in the evolutionary field at the best possible seasonal quality factor Q[n] for each agent. (A proof of this proposition is given with Remark A2 in Appendix A.) All agents perform a geometrical crossover at the highest quality factor toward the seasonal best code $X_{best}\left[n\right]$, and thus maximize the total quality ($\sum_{i=1}^{h_{k}}Q_{i}\left[n\right]$) in each seasonal evolution. Then, a field-adapted differential crossover is defined according to the following quality function-based evolution rule:

(11) $$x_{p,i}\leftarrow x_{p,i}+\left(x_{best,i}-x_{p,i}\right)\frac{\left|F\left(X_{best}\left[n\right]\right)-F\left(X_{p}\left[n\right]\right)\right|}{\left|F(X_{best}\left[n\right])\right|+\left|F(X_{p}\left[n\right])\right|}$$

This field-adapted differential crossover is applied to the property code of agents except the best agent $X_{best}\left[n\right]$. The best agent $X_{best}\left[n\right]$ performs field-aware metamutation. The evolutionary field values of agents are measured by the objective function according to the performance of all agents in a solution environment. The seasonal best agent of the evolutionary field is found by $X_{best}\left[n\right]=\underset{X_{j}}{argmin}F\left(X_{j}\left[n\right]\right)$. Then, the field-adapted differential crossover update of the code property in the evolutionary field can be expressed as

(12) $$p_{c}=\left(x_{best,i}-x_{p,i}\right)\frac{\left|F(X_{best}\left[n\right]-F\left(X_{p}\left[n\right]\right)\right|}{\left|F(X_{best}\left[n\right])\right|+\left|F(X_{p}\left[n\right])\right|}.$$

Such an update of *i*th property of agents enables the transformation of agents with their experience in the solution environment toward the more successful agent properties with the highest total seasonal quality factor.

(ii) *Field-aware mutation and bifurcated metamutation of property codes:* To gain a field awareness in the mutation process, the mutation process is performed depending on the field value $F(X_{k}\left[n\right])$. Thus, the evolution tendency of agent properties is regulated according to their field values. More mutation tendency is promoted for agent property codes that suit the solution environment less because their field values are high. This regulation results in a field awareness in the mutation process. This behavior is closely related to the conjecture that increased difficulty in living conditions and rise in environmental stresses can lead to more coincidental mutation of living organisms in nature, and such increase in the mutation tendency, in turn, contributes to search of more suitable characteristic properties to manage adaptation of the organism to the environment. In this mutation rule of the proposed algorithm, less fitting agents of the solution environment should exhibit more tendencies for mutating in the evolutionary field, and this leads to more exploration in the evolutionary field. The seasonal field-aware mutation tendency of an agent property is expressed relative to the best agent of the evolutionary field as

(13) $$p_{g}=\frac{F(X_{p})}{F\left(X_{p}\right)+F\left(X_{best}\right)}.$$

The mutation update of agent properties in the field should be a stochastic process to enrich search possibilities; the field-aware mutation update is expressed as

(14) $$P_{m}=r_{m}p_{g}=r_{m}\frac{F(X_{p})}{F\left(X_{p}\right)+F\left(X_{best}\right)}.$$

where the parameter $r_{m}$ is a uniform random number in a range of [−0.5, 0.5] and $p_{g}$ stands for the field-aware mutation range. This equation provides a randomization in mutation of the agent properties in a range that is relative to the field value of the best agent property. When the field value of the best agent decreases to a lower field value, other agents tend to perform more mutations to become more competitive in exploration. If the best agent does not reach low field values, the other agents become more conservative by reducing their mutation range $p_{g}$ to perform more exploitation in their local property space.

After introducing the field-adapted differential crossover and field-aware mutation process of the property codes, the next-generation agent property in the field is produced by aggregation of contributions of the crossover and mutation processes to the property code in each season. The property code update rule of an agent can be written as a linear combination of updates of these genetic processes.

(15) $$x_{p,i}\leftarrow x_{p,i}+c_{1}p_{c}+c_{2}p_{m}$$

However, the best agent property does not perform the field-adapted differential crossover and the field-aware mutation as other search agents. To gain more awareness on the field topology, the best property code is rewarded to mutate around the field center of all property codes. This metamutation behavior considers the constellation of other code properties and benefits from the geometrical knowledge associated with the center of property code distribution. This knowledge is extracted by calculating the reverse-weighted center of the code constellation that is expressed as

(16) $$x_{o,i}=\left(1-\frac{F(X_{j})}{\sum_{l=1}^{h_{k}}F\left(X_{l}\right)}\right)x_{j,i}.$$

The reverse-weighted center is a formulation of the behavior that the property code with lower field value has higher weight in the determination of the center point. (Roulette wheel selection method of the genetic algorithm uses a similar formulation for its reproduction process.) Since bifurcated metamutation of the best property code around a global property center and a personal property center is useful to increase exploration potential of the best property code, we performed a bifurcated metamutation by selecting one of two processes with equal probability: the first one is the search of regions around the reverse-weighted center in a range of a scattering radius $A_{scat}$ of the other agent code constellations in the field. The mutation in this field region is called property assimilation. The second is the random exploration around its own surrounding property code space with a scattering radius $A_{scat}$. This mutation region is called property conservatism. The scattering radius of the code constellation is determined by the absolute scattering radius of agent constellations in the evolutionary field. Finally, the best property code can produce a new code according to

(17) $$x_{best,i}\leftarrow \left\{\begin{array}{cc} x_{o,j}+r_{b}p_{out}A_{scat} & rand\leq 0.5\\ x_{best,j}+r_{b}p_{out}A_{scat} & rand>0.5 \end{array}\right\},$$

where rand is a uniform random number in the range of [0, 1]. The parameter $r_{b}$ is a random number in the range of [−0.5, 0.5] to randomize the search in these regions. The $p_{out}$ is a weight coefficient to resize the absolute scattering radius $A_{scat}$. The absolute scattering function $A_{scat}$ is calculated as

(18) $$A_{scat}=\frac{1}{h_{k}}\sum_{j=1}^{h_{k}}\left|X_{j}-X_{avg}\right|,$$

(19) $$X_{avg}=\frac{1}{h_{k}}\sum_{j=1}^{h_{k}}X_{j}.$$

**Figure 5:**
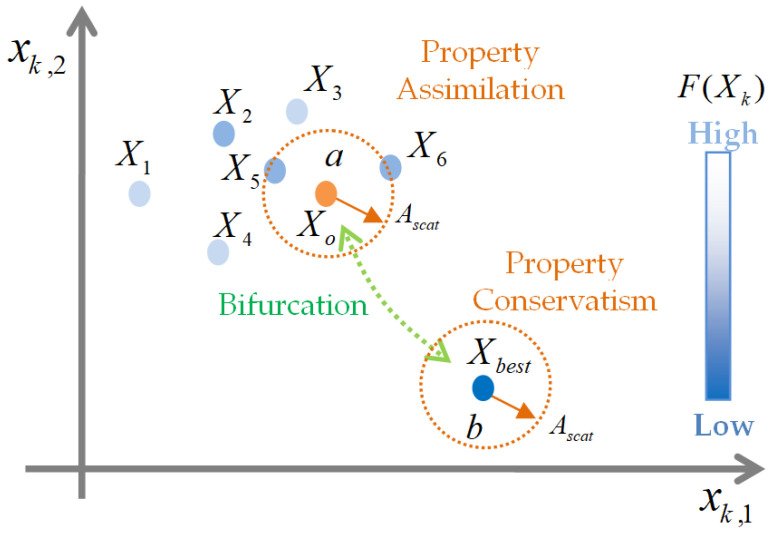
Depiction of two search areas of the best agent property code in a two-dimensional evolution field.

The term of metamutation was previously used in several previous works in a different context, to define mutation process improvements [59,60,61]. However, these definitions are not the same with the concept of bifurcated metamutation process that is defined in the current study. Figure 5 shows a depiction of two search areas of metamutation of the best property code. These regions are indicated by a dashed circle with the center *a* for the property assimilation and a dashed circle with the center *b* for the property conservatism. As a result, the best agent property code repositions with a probability of 0.5 in the field space with reverse-weighted center $X_{0}$ (a preference of property assimilation to survive among other successful agents) or its own position $X_{best}$ and the radius of $A_{scat}$ (a preference of property conservatism to survive with its own possessions). Steps of the EFO-GS algorithm can be summarized as follows:

Step 1 Randomly distribute all agent property codes $X_{k}$ within the evolution field;
Step 2 Calculate the field values $F(X_{k})$ for each property codes $X_{k}$;
Step 3 Select the seasonal best agent property code as $X_{best}\left[n\right]=\underset{X_{j}}{argmin}F\left(X_{j}\left[n\right]\right)$;
Step 4 Perform the field-adapted differential crossover and field-aware mutation combination for agent property codes according to Equation (15) except the seasonal best agent property code $X_{best}$ and obtain new generation candidates of the seasonal property codes, $\tilde{X_{k}}$;
Step 5 Perform only bifurcated metamutation for the seasonal best agent property code $X_{best}$ according to Equation (17) and obtain a new generation candidate of the seasonal property code ${\tilde{X}}_{best}$;
Step 6 Form a seasonally evolved new generation set of the seasonal property codes as ${\tilde{X}}_{k}=\left\{{\tilde{X}}_{best},{\tilde{X}}_{k}\right\}$, and calculate the field values $F({\tilde{X}}_{k})$ for each new generation property codes from ${\tilde{X}}_{k}$;
Step 7 Select the agent property codes with lower field values from old and new property code collections $\left\{X_{k},{\tilde{X}}_{k}\right\}$ and update the set of $X_{k}$;
Step 8 If a predefined stopping criteria is not met, select the best agent property code with the lowest field value as the optimal solution of the optimization problem. Otherwise, go back to step 3.

## 3. Evolutionary Training of Power-Weighted Multiplicative Neural Processor via Evolutionary Field Optimization Algorithm

Evolutionary optimization methods have been used in the training of artificial neural networks [7,12,62,63], and this training method is known as evolutionary training. Primarily, Whitley et al. used a genetic algorithm in weight optimization of neural network by using binary encoding of weights [62] and the weight coefficients of the neural network are represented by a string of binary values. This causes a limitation for weight values because the expression precision of binary encoding may not be enough for every application [7]. Then, the real number encoding was used to express weights in the genetic algorithm [64]. Some works reported that training performances of GA were comparable with the training of backpropagation method because the backpropagation method uses a gradient-based local search and it may easily fall into local minimums [13]. Evolutionary training methods can perform a global search strategy and this may improve the search performance compared to a local search. However, the number of optimized parameters (dimension of search space) can limit the performance of the evolutionary methods due to exponential growing of the search space. Xiangping et al. showed that a hybridization of GA and backpropagation methods can improve the training performance, where the GA determines optimal initial values of weights for the backpropagation algorithm [65]. In the literature, several Evolution Algorithms (EAs) have been used for the training of ANNs [24,66,67] and performance improvements and shortcomings were discussed. In the current study, an EFO training of the suggested power-weighted multiplicative neural processor will be carried out and an application in electronic nose design for NO_x_ measurement and control for the aerospace industry and air quality is presented in the following sections.

Electronic noses have been widely utilized for detection and classification of gases by implementing machine learning classifiers [68,69]. They have also been used for accurate measurement of gas concentrations by using machine learning-based sensor calibrators [70,71,72]. Today, electronic nose technologies can contribute to the improvement of many daily-life processes. For instance, the gas sensors and electronic nose solutions promise important agricultural applications; for instance, monitoring and prediction of important parameters related to the growth and harvest of a crop, and allow data-driven management practices in several stages of agricultural activities [73]. Another useful application of electronic noses was demonstrated for discrimination of pathogenic bacterial volatile compounds [74].

### 3.1. Preliminaries for Multiplicative Unit

The addition of multiplicative units to the classical neuron model of McCulloch and Pitts [36] has some origins in biological research studies [47,49] and mathematical studies [46,49]. First of all, multiplicative units can increase the nonlinear approximation and representation capabilities of neural networks [46,49]. Some works, which implemented multiplication in neural processing, have suggested that the use of polynomial nonlinearity [52,53], power series, and Binomial series [54] in the classical neuron model contributes to approximation skills of neuron models compared to classical neuron models. Effects of product units in neural processing have been also elaborated in several preliminary works [47,48,49]. The multiplicative unit (product term) was defined as

(20) $$u=\prod_{j}^{}x_{j}^{p_{j}}.$$

where the input variable $x_{j}$ is a positive real variable and the power (exponent) $p_{j}$ is a positive real number [48,51]. It is very useful to consider Simon’s discussion to gain deeper insight on relations between that multiplicative neural networks and additive neural networks [51]. Simons revealed the fact that the multiplicative neural network can be expressed in the form of an additive network with a different nonlinearity formulation that originates from the identity $\prod_{j}^{}x^{p_{i,j}}=e^{\sum_{j}^{}p_{i,j}In\left(x_{j}\right)}$ [47,51]. This was a very important and useful observation from a machine learning point of view because it opens a door for implementation of multiplicative elements in neuron networks similar to additive elements. In this section, we extend this discussion and consider the relation between multiplicative units and the weighted geometric average to observe some assets. The multiplicative unit, which is defined by Equation (20), is a generalization of the weighted geometric average operator, which has been solely utilized in the calculation of multiplicative preference or priorities in decision making [75,76,77,78]. The multiplicative unit turns into a weighted geometric average operator when the normalization condition $\sum_{j}^{h}p_{j}=1$ is satisfied (see Remark A3 in Appendix A). In essence, the multiplication of variables can be useful to process exponential relations between the parameters. Another advantage may be that the multiplication operator allows more spread of results over a wider value set than the addition operator because multiplication of parameters is mostly greater than addition of those parameters in many cases. Essentially, additive units can perform the weighted sum operation that presents correspondence with the arithmetic average.

### 3.2. Power-Weighted Multiplicative Neural Processing

**Figure 6:**
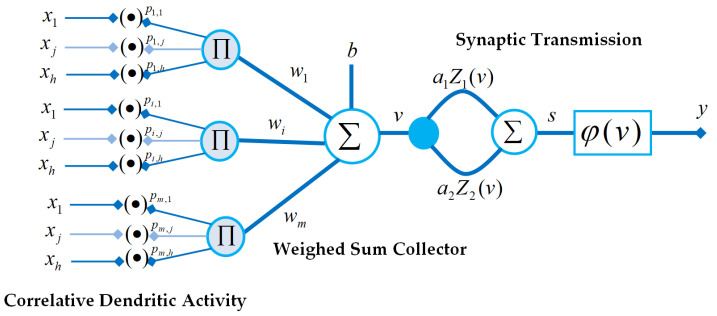
A block diagram of essential functional blocks of the power-weighted multiplicative neuron model.

This section introduces a general formulation of the power-weighted multiplicative neuron model for artificial neural processing of data. Figure 6 shows a block diagram that represents essential functional blocks of this neuron model. There are two additional functional blocks that are appended to the classical neuron model suggested by McCulloch and Pitts [36].

A power-weighted multiplication operator is used to represent the dendritic activity in the PWM neuron model. From the neurobiology origin, Kerlin et al. stated that active properties of dendrites can support the local nonlinear operations in the neuron functioning [79] and this is an important effect for processing real-world stimulus. The dendritic activity is not explicitly considered in the classical neuron model as a separate function; instead, both nonlinearity effect and output value limiting effect are performed by designing suitable activation functions. We used the power-weighted multiplication operations in order to represent nonlinear relations in unification of dendritic branches. The power-weighted multiplication is expressed to process neuron inputs $x_{1},x_{2},x_{3},\ldots ,x_{h}$ as

(21) $$u_{i}=\prod_{j=1}^{h}x_{j}^{p_{i,j}}.$$

where the power weight $p_{i,j}$ represents *j*th input (branch) of *i*th dendritic branch in the neuron. The parameter *h* is the number of inputs in the neuron model. Then, results of dendrites are collected by using a weighted sum operation and adding a bias *b* as follows:

(22) $$v=\sum_{i=1}^{m}\left(w_{i}u_{i}\right)+b$$

where the parameter $w_{i}$ is the weight of *i*th dendritic branch. Due to the fractional power of inputs, the PWM neural model can produce complex numbers, and accordingly, it can work as a complex-valued neuron. To see this operation, let us assume a negative input x<0 and a fractional power $p_{r}\in R$ that is a non-integer number $p_{r}\notin Z$, one can write (see Proposition A1 in Appendix A)

(23) $$x^{p_{r}}={\left|x\right|}^{p_{r}}\left(cos\left(\pi p_{r}\right)+jsin\left(\pi p_{r}\right)\right).$$

**Figure 7:**
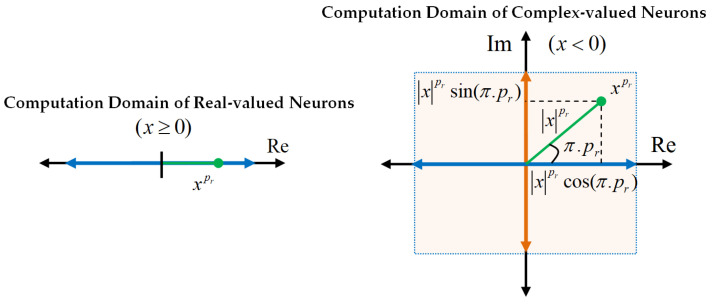
The domain of the real-valued neuron and the domain of the complex-valued neuron and related properties.

Equation (23) clearly shows that a negative-valued input $x_{j}<0$ and a fractional power weight $p_{i,j}\in R-\left\{Z\right\}$ result in a complex value $u_{i}\in C$. Consequently, the PWM neural processor can perform in the complex–valued neuron mode. Advantages of the complex-valued neural neurons have been comprehensively reviewed by Bassey et al. [80]. In this comprehensive review work, contributions of the additional phase information to neural learning process have been highlighted. In order to contribute to this discussion in the current study, Figure 7 illustrates the domain of real-valued neurons and the domain of the complex-valued neurons. The figure clearly demonstrates the co-domain expansion of the neural function from a one-dimensional line into a plane by means of processing the complex values. The complex signal properties associated with this domain expansion (e.g., real and imaginary components, magnitude and phase properties) are also shown in the figure. Such expansion to the complex number domain can enhance data processing skills of PWM neurons because the complex-valued operation zone already covers its real-valued counterparts in the computation task.

As summary, one can observe that the proposed PWM neuron model can operate as the real-valued neuron, the complex-valued neuron, and mixed-mode neuron depending on the interval of input values. Table 2 lists operation modes of a PWM neuron. To switch operation modes of the neuron to the complex-valued or the real-valued modes, an interval shifting scheme is suggested for the set of the input *x*.

Due to the complex-valued operations of the power-weighted multiplicative neural processing, we added a mapping-to-real function to obtain real-valued outputs. The mapping-to-real functions map the weighted sum of dual complex number properties to a real value, which enables to convert results of neural processing in complex number domain to a real-valued signal for transmission of a real signal to the neuron output. A generic mapping-to-real function is defined as

(24) $$s=a_{1}z_{1\lambda }\left(v\right)+a_{1}z_{2\lambda }\left(v\right),$$

where $z_{1\lambda }\left(v\right)$ and $z_{2\lambda }\left(v\right)$ are dual property functions, and parameters $a_{1}$ and $a_{2}$ are corresponding weights of dual properties of complex numbers (e.g., real-imaginary properties or magnitude-phase properties). Complex numbers have two types of dual properties that can be implemented by $z_{1\lambda }\left(v\right)$ and $z_{2\lambda }\left(v\right)$ functions with λ=c,p. These are

(i) Cartesian (λ=c) properties: real and imaginary parts of the complex number $v=v_{r}+jv_{im}$:
  (25) $$z_{1c}\left(v\right)=Re\left\{v\right\}=v_{r}\;\mathrm{and}\;z_{2c}\left(v\right)=Im\left\{v\right\}=v_{im}$$
(ii) Polar (λ=p) properties: magnitude and phase properties of the complex number $v=v_{r}+jv_{im}$:
  (26) $$z_{1p}\left(v\right)=\sqrt{v_{r}^{2}+v_{im}^{2}}\;\mathrm{and}\;z_{2p}\left(v\right)=tan^{-1}\left(\frac{v_{r}}{v_{im}}\right)$$

If parameters $a_{1}$ and $a_{2}$ are determined during the training process, the mapping-to-real function contributes to the learning process and performs a trainable mapping. However, they can be set to fixed values to gain desired properties for the neuron. For example, for polar properties, setting $a_{1}=1$ and $a_{2}=0$ results in a mapping depending on the magnitude of the complex number and it yields a positive real number. In addition, the mapping according to the phase information can be obtained by setting $a_{1}=0$ and $a_{1}=1$.

Following the mapping-to-real function, an activation function can be used to the limit output values of the PWM neuron to predefined output ranges. This may represent the limited amplitude signals in the synaptic transmission of biological neurons. Well-known activation functions φ(s) is a linear activation function to avoid any change, the sigmoid activation to limit the output in a range of [0, 1] or the tangent hyperbolic activation to limit the output in a range of [−1, 1]. Other popular activation functions or parametric activation functions can be used. The output of a neuron is written as

(27) y=φ(s).

This neuron model can be a generalization of other artificial neuron models and it is capable of expressing several well-known models after properly selecting the PWM neuron model parameters. Table 3 shows representation of several neuron models that are obtained by suitable selection of PWM neuron model parameters. Therefore, special cases of the PWM neuron model can express other neuron models in Table 3. This reveals the fact that the model representation capacity of the PWM neuron model covers these models. Such a model coverage enhancement leads to additional parameters to optimize and the associated training difficulties. Therefore, neuroevolutionary approaches and metaheuristic methods can be preferable in the training of this type of sophisticated neural model. In the current study, the proposed EFO-GS algorithm is implemented to perform the evolutionary training of PWM neural models.

Let us express the overall network function of PWM neurons. To consider a complex-valued neuron, which is the general case in operation of a PWM neuron, one can rewrite Equation (22) by using Equation (23) in Equation (21) as (see Theorem A1 in Appendix A)

(28) $$v=b+\sum_{i=1}^{m}\left(w_{i}\left(\prod_{k=1}^{h}{\left|x_{k}\right|}^{p_{i,k}}cos\left(\pi \sum_{l=1}^{h}p_{i,l}\right)\right)\right)+j\sum_{i=1}^{m}\left(w_{i}\left(\prod_{k=1}^{h}{\left|x_{k}\right|}^{p_{i,k}}sin\left(\pi \sum_{l=1}^{h}p_{i,l}\right)\right)\right).$$

The real and imaginary parts of $v=v_{r}+jv_{m}$ are obtained as

(29) $$z_{1c}=v_{r}=b+\sum_{i=1}^{m}\left(w_{i}\left(\prod_{k=1}^{h}{\left|x_{k}\right|}^{p_{i,k}}cos\left(\pi \sum_{l=1}^{h}p_{i,l}\right)\right)\right),$$

(30) $$z_{2c}=v_{im}=\sum_{i=1}^{m}\left(w_{i}\left(\prod_{k=1}^{h}{\left|x_{k}\right|}^{p_{i,k}}sin\left(\pi \sum_{l=1}^{h}p_{i,l}\right)\right)\right).$$

Then, the mapping-to-real function for Cartesian properties (λ=c) yields

(31) $$s=a_{1}b+a_{1}\sum_{i=1}^{m}\left(w_{i}\left(\prod_{k=1}^{h}{\left|x_{k}\right|}^{p_{i,k}}cos\left(\pi \sum_{l=1}^{h}p_{i,l}\right)\right)\right)+a_{2}j\sum_{i=1}^{m}\left(w_{i}\left(\prod_{k=1}^{h}{\left|x_{k}\right|}^{p_{i,k}}sin\left(\pi \sum_{l=1}^{h}p_{i,l}\right)\right)\right).$$

The mapping-to-real function for polar properties (λ=p) is calculated as

(32) $$z_{1p}=\left|v\right|=\sqrt{\left(v_{r}^{2}+v_{im}^{2}\right)},$$

(33) $$z_{2p}=arg\left(v\right)=tan^{-1}\left(\frac{v_{im}}{v_{r}}\right),$$

(34) $$s=a_{1}\left|v\right|+a_{2}arg\left(v\right).$$

These solutions reveal the following remarks:

- When $\sum_{k=1}^{h}p_{i,k}\in Z$ or $\forall p_{i,k}\in Z$, then it results in $sin(\pi \sum_{l=1}^{h}p_{i,l})=0$ and $cos\left(\pi \sum_{l=1}^{h}p_{i,l}\right)={\left(-1\right)}^{\sum_{l=1}^{h}p_{i,l}}$, the PWM neuron operates in the real-valued mode, and its function can be simplified to
  (35) $$s=a_{1}b+a_{1}\sum_{i=1}^{m}\left(w_{i}\left(\prod_{k=1}^{h}{\left|x_{k}\right|}^{p_{i,k}}{\left(-1\right)}^{\sum_{l=1}^{h}p_{i,l}}\right)\right).$$
- When $\sum_{k=1}^{h}p_{i,k}\notin Z$ or $\exists p_{i,k}\notin Z$, a PWM neuron operates in the complex-valued mode as shown by Equation (28).

## 4. Experimental Study

### 4.1. An Electronic Nose Application for Monitoring NO_x_ Concentration by Solid-State Multisensor Array

Artificial neural networks have wider utilization in machine learning when computational intelligence with learning ability is essential for applications. Data-driven control of complex real systems is becoming a central topic within the machine learning application domain because of promising intelligent real-world systems. In today’s intelligent system concepts, artificial neural networks preprocess the fused data stream from sensor networks, and they can provide reliable estimation of the current and future system states and they may produce suitable control responses to regulate the monitored system status. Inevitably, the preservation of the air quality in crowded cities requires an active, data-driven air quality control scheme that can detect local buildups of pollutants in urban areas. One of the important atmospheric pollutants is nitrogen oxide (NO_x_). Monitoring of NO_x_ emission has been considered to preserve air quality in crowded cities [70], to increase fuel efficiency in NO_x_ emissions in aviation and aerospace industry [81], to improve design of gas turbine engines for aircraft and power stations [82]. Due to the large size and high cost of chemical analyzers, low-cost solid state sensor arrays have begun to be utilized in on-field monitoring of pollutant gases [70,71,83,84]. However, measurements of low-cost multisensor arrays are not accurate, and they need calibration according to the precise measurements of chemical analyzers. Therefore, an artificial neural network was implemented to estimate chemical analyzer measurements from measurements of the multisensor arrays, and the effectiveness of this soft-calibration approach (calibration by software) was shown in an air-quality monitoring application [71]. A cooperation of multisensor arrays with measurement systems is referred to as an electronic nose system. The estimation model performs for the sensor calibration in order to improve precision of measurements [70,71] and machine learning-based sensor calibration was preferred for intelligent systems. Then, the soft-calibration models have become an essential part of electronic nose systems. The current experimental study shows implementation of the PWM neural processor with EFO-GS as the soft-calibration model. A PWM neural model was trained for accurate estimation of NO_x_ concentrations from a low-cost multisensor array measurement dataset. This dataset includes hourly measurements from solid state gas sensors, commercial temperature and humidity sensors, and a conventional air pollution analyzer (the reference chemical analyzer was used for the ground truth data) [70,71]. A microcontroller board, which was hosting a microprocessor, a GSM (Global System for Mobile Communications) data transmission unit, and the solid state sensor array, was used to collect sensor data with a sampling period of 8 s, and an hour average of the sensor data was used to form hourly measurement instances in the dataset [70,71]. Table 4 introduces these sensors and calibrator model parameters. The training dataset was composed of 586 measurement instances that were collected during 24 days, and the following 241 measurement instances were used for the test dataset in order to estimate the next 10-day-long hourly measurements.

To implement EFO-GS algorithm for the training of PWM neural network, the property code of the EFO-GS includes all coefficients of the single PWM neuron model as

(36) $$X_{k}=\left[\begin{array}{ccccccc} W_{k} & b_{k} & P_{k,1} & P_{k,2} & \ldots & P_{k,m} & A_{k} \end{array}\right]$$

where weight coefficients of the sum unit are denoted by $W_{k}=\left[w_{k,1}\;w_{k,2}\;\ldots \;w_{k,m}\right]$, the coefficients of the power-weighted multiplication in *i*^th^ dendritic branch are represented by $P_{k,i}=\left[p_{k,i,1}\;p_{k,i,2}\;\ldots \;p_{k,i,h}\right]$, and coefficients of generic mapping-to-real function are $A_{k}=\left[a_{k,1}\;a_{k,2}\right]$. The EFO-GS algorithm minimizes the sum of the square loss function to perform training of the PWM neuron model.

**Figure 8:**
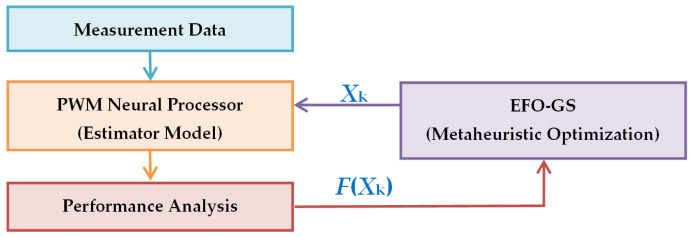
This flowchart describes employment of the EFO-GS algorithm for training of PWM neural processor in order to obtain a data-driven estimation model.

**Figure 9:**
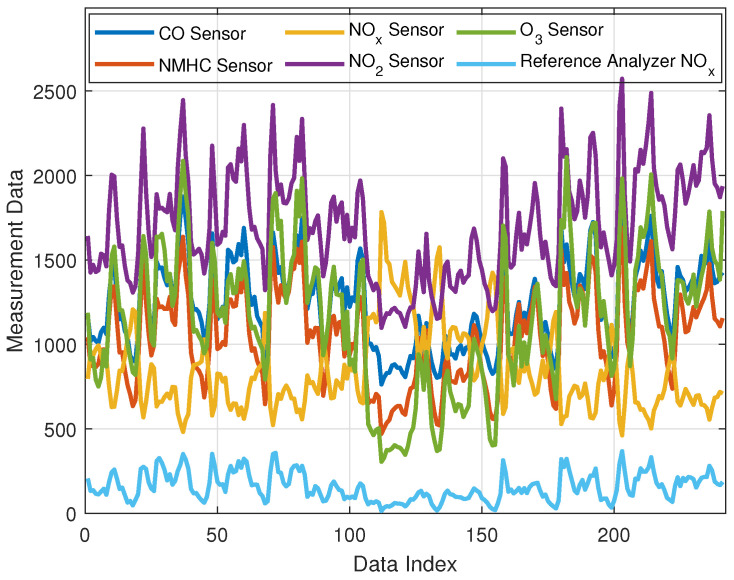
Measurements data of low-cost sensory array that are sensitive to CO, NMHC, NO_x_, NO_2_, and O_3_ molecules and the reference chemical analyzer measurement for NO_x_.

Figure 8 shows a flowchart that describes implementation of the EFO-GS algorithm for training of PWM neural processors in order to obtain an estimation model from measurement data. This chart is also a general block diagram of metaheuristic data analysis scheme where the PWM neural model is a learning model from the dataset, and the metaheuristic optimization is used to solve the optimization problem in order to find an optimal solution of the data analysis problem. This application indeed solves a measurement error reduction problem (a soft-calibration problem) for on-field sensor data. Figure 9 shows 241 measurement instances that are hourly averages of the multisensor array data and the reference chemical analyzer measurements for NO_x_. The figure illustrates the test dataset that includes the collected data from solid state multisensors sensitive to CO, NMHC, NO_x_, NO_2_, O_3_, and the reference chemical analyzer for NO_x_ during 10 days of observation (*y*-axis shows the values of average concentration measurements from sensors and the chemical analyzer, and *x*-axis indicates the measurement instances). The reference chemical analyzer measurements are correct measurements to be learned by machine learning methods to calibrate low-cost sensor arrays.

To show modeling performance of a PWM neuron, a single PWM neuron with 8 inputs and 5 dendrites was implemented in the real-valued mode, and its performance was compared with a multi-layer classical ANN model and a Genetic Programming (GP) model. The training dataset is used to obtain an estimation model in the form of $y_{d}=f\left(x_{1},x_{2},x_{3},x_{4},x_{5},x_{6},x_{7},x_{8}\right)$.

**Figure 10:**
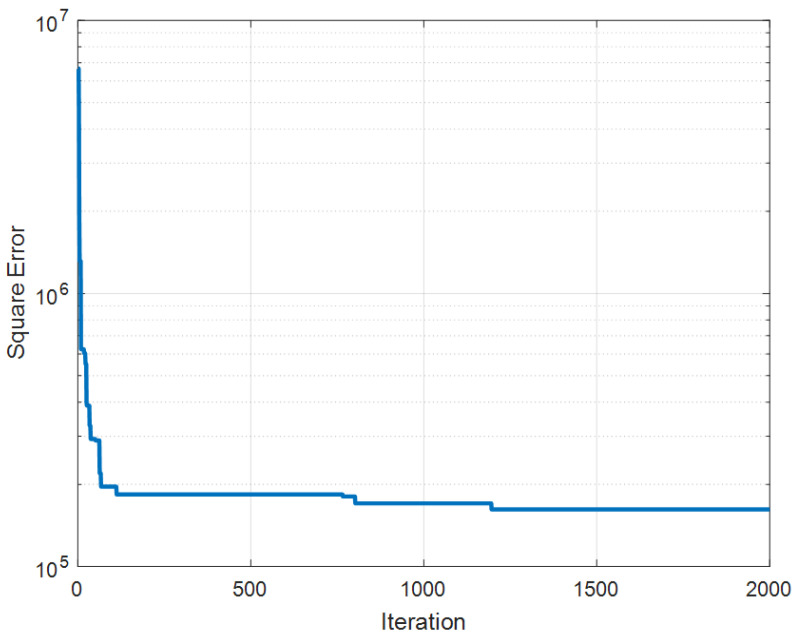
Convergence of the square error during the EFO-GS optimization of the PWM neuron for NO_x_ concentration estimation.

Figure 10 shows the convergence of square error during EFO-GS optimization of the single PWM neuron for the NO_x_ concentration estimation model. The EFO-GS has performed 2000 iterations and optimizes 48 parameters of the PWM neuron. (The number of fractional power weight $p_{i,j}$ is 8 × 5 = 40 parameters, the number of weights $w_{i}$ for five dendritic branches is 5, the number of bias (*b*) is one and number of mapping-to-real function parameters (a1,a2) is 2.) Training tasks were performed for 586 hourly measurement data and performance tests were performed for the subsequent 241 hourly measurement data in Figure 9. The test data were not involved in any stage of the training of the PWM neuron. The classical neural network was implemented with 3 layers. It has 10 neurons in the first hidden layer, 2 neurons in the second hidden layer, and one neuron at the output layer. The total number of weight parameters is 115. The GP model was implemented by using the GP algorithm with Orthogonal Least Square (GpOls) [85]. The GpOls algorithm was developed for effective identification of nonlinear input–output models by using a tree-based genetic programming with a linear least square modeling technique [85].

**Figure 11:**
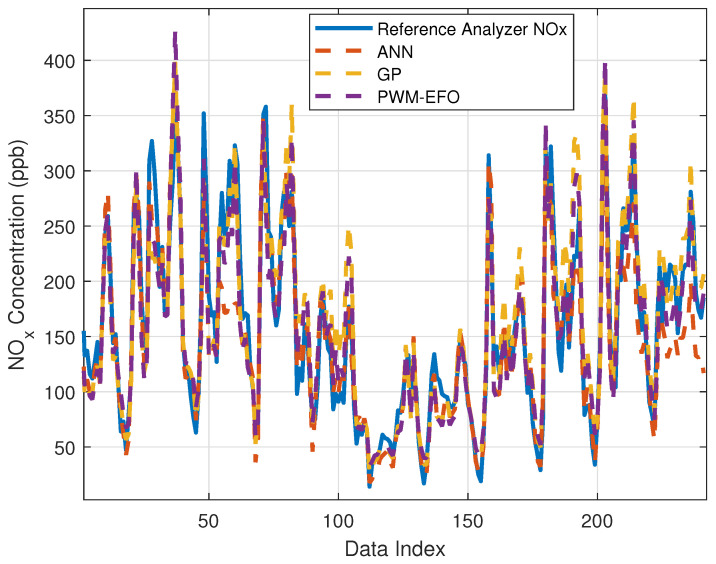
Estimations of ANN, GP, PWM-EFO for the test dataset.

**Figure 12:**
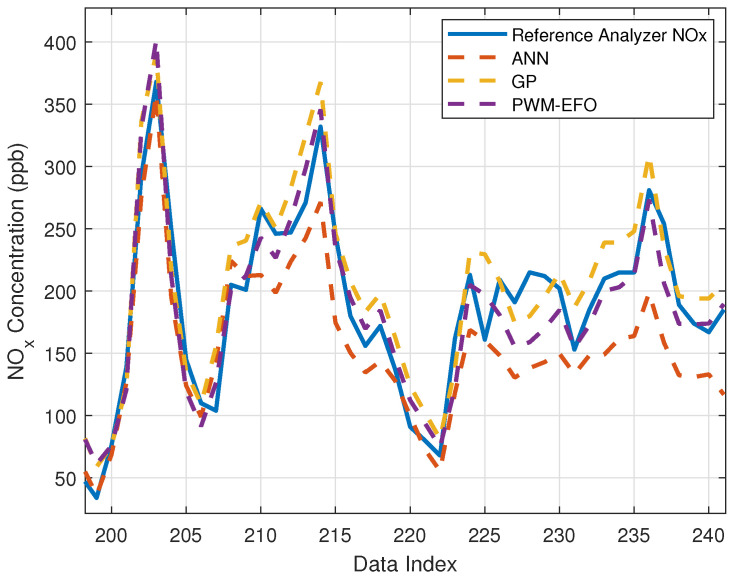
A close view of concentration estimations between 200th hour and 240th hour in Figure 11.

Figure 11 shows NO_x_ concentration estimations of the tested machine learning methods for the test dataset. To better view convergence of the estimation model to the reference analyzer measurements (ground truth measurements), Figure 12 presents a close view of Figure 11. The figures reveal that all estimation models provide consistent estimates, and these models can be used for the calibration of multisensor arrays in practice. However, the PWM neuron uses quite less optimization parameters than the ANN model to reach this performance level.

**Figure 13:**
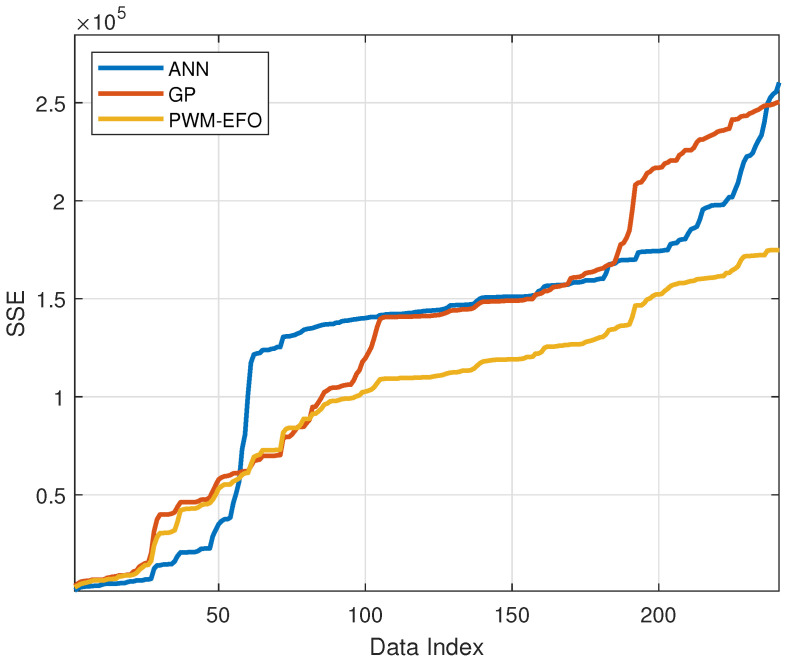
Increase of sum of square error (SSE) during estimations for three models.

Table 5 lists performance indices in order to evaluate concentration estimation performances of the PWM neuron with EFO-GS, classical ANN, and GP models for NO_x_ measurements. Regression performance is widely evaluated by using Mean Square Error (MSE). MSE performance of the PWM neuron with EFO-GS is better than other models. The R^2^ score measures the fitting performance of models to data in the regression analysis. The PWM neuron with EFO-GS provides an R^2^ score of 89%, which is higher than that of the other models. Figure 13 shows change of the sum of square error (SSE) through the estimation period, and it evaluates the cumulative square error distribution for all test data. In the beginning, the SSE performance of ANNs is better. However, after a 50-h estimation period, the square error of ANN model sharply increases. The SSE model of GP model exhibits an instant SSE rise around 100 h. The PWM neuron with EFO-GS is more consistent for long-term estimation, and this indicates that data generalization of the PWM neuron with EFO-GS can be better than other methods. It is useful to consider the histogram of instant measurement errors to validate this effect.

**Figure 14:**
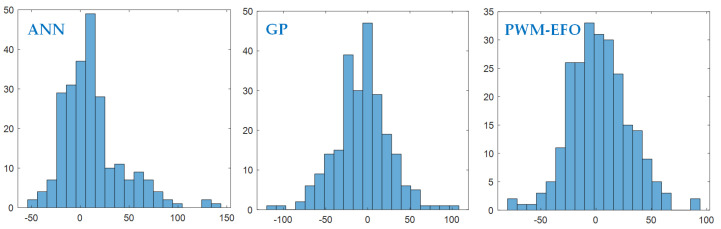
Distribution of instant measurement errors for three models.

Figure 14 shows histogram analysis of estimation errors according to the reference analyzer measurements. These figures illustrate a distribution of instant measurement errors around zero value. The measurement error distribution of the PWM-EFO indicates more successful estimation and generalization from the training dataset so that instant errors accumulate near to zero and distribution around zero is more balanced and similar to the normal distribution. Results in Table 6 confirm observations in histogram analysis. For successful estimation and generalization, the mean value of estimation errors should be zero, the standard deviation of estimation errors should be minimum, and distribution around zero be more balanced (symmetrical) for the test data. This implies that useful modeling information is absorbed from the training dataset. The instant measurement error of the PWM-EFO has a mean value that is closer to zero, which indicates an improved generalization of data, and it has the lowest standard deviation, which implies better learning from the data.

### 4.2. Experimental Results for Complex-Valued Mode PWM-EFO Neuron

**Figure 15:**
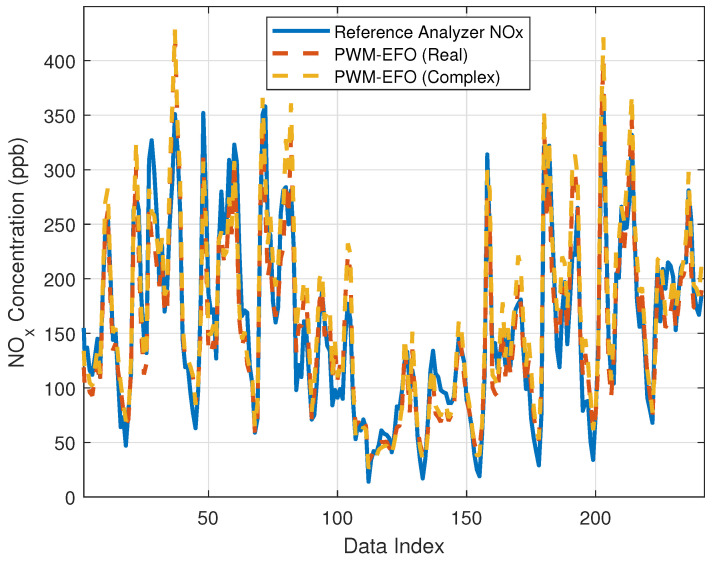
Estimations of the real-valued mode PWM neuron and the complex-valued mode PWM neuron for the test dataset.

This section presents the results of the real-valued neuron mode and the complex-valued neuron mode of the PWM-EFO method for NO_x_ estimation. The complex-valued mode was activated by assigning a negative sign to the multisensor array input data that were originally all positive-valued. This makes all input values a negative real number, and the dendritic branches of PWM yield complex numbers according to Equation (23). The PWM neuron processes complex numbers. Accordingly, the training dataset was arranged in the form of ($-x_{1},-x_{2},-x_{3},-x_{4},-x_{5},-x_{6},-x_{7},-x_{8},y_{d}$) to shift in the complex-valued neuron mode. In the previous section, it worked in the real-valued PWM neuron mode since the training dataset was arranged in the form of ($x_{1},x_{2},x_{3},x_{4},x_{5},x_{6},x_{7},x_{8},y_{d}$). Figure 15 shows estimations of these PWM neuron modes for the test dataset. Table 7 shows estimation performance indices. Results indicate that the estimation performance of the real-valued mode operation is slightly better than those of the complex-valued mode. A reason for these results can be that the dataset has a nature of real-valued relations, and there may be no need for computation in the complex domain for this dataset.

**Figure 16:**
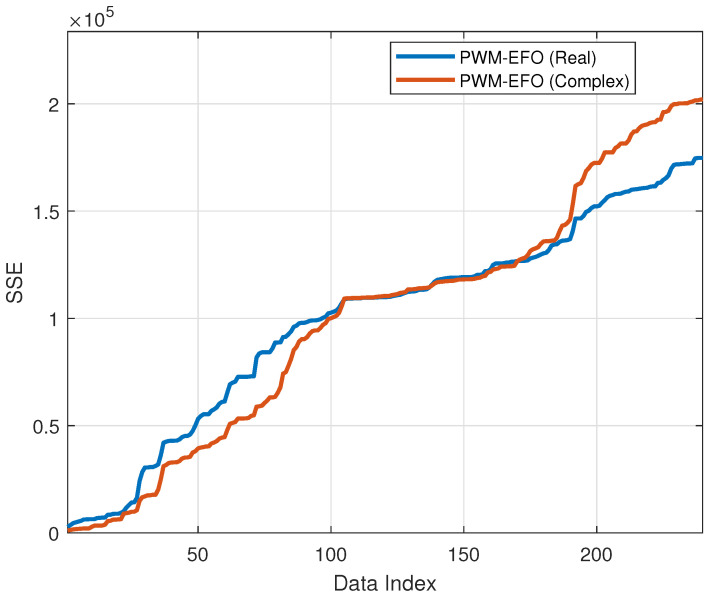
Increase of sum of square error (SSE) during estimations via the real-valued mode PWM neuron and the complex-valued mode PWM neuron.

Figure 16 shows the change of the sum of square error (SSE) during 241 hourly measurement estimations while using real-valued mode and complex-valued mode PWM neurons. Up to the 100th measurement, the complex-valued mode provides a better SSE performance; however, around the 190th measurement, its SSE performance is getting worse. Overall, the long-term SSE performance of real-valued neurons is better, and these results indicate that the generalization of the training dataset is better for the real-valued mode in this NO_x_ calibration problem.

## 5. Conclusions

This study suggested an evolution field theorem to establish a theoretical background for the analysis of the agent-based evolutionary computation systems and an EFO-GS optimization algorithm was introduced on the basis of this theorem. This algorithm performs a geometrical evolution according to the evolutionary field values under the assumption of a Markovian search process. The evolution field theorem can form a common theoretical basis, where population-based evolutionary optimization algorithms can be analyzed, designed, and compared. Another contribution of this study addressed the improvement of basic neuron models: after briefly reviewing multiplicative neuron studies, the computational scheme of the multiplicative neurons were modified by using non-integer power weights and the mapping-to-real function block. Thus, a PWM neural processing unit with multi-mode operation was suggested as a generalization of classical ANNs. The EFO-GS optimization was implemented for the training of the PWM neurons. Operation modes of the suggested PWM neurons were investigated in detail, and computational supremacy of the PWM neurons over conventional neural models was discussed theoretically and shown experimentally in the electronic nose application.

**Figure 17:**
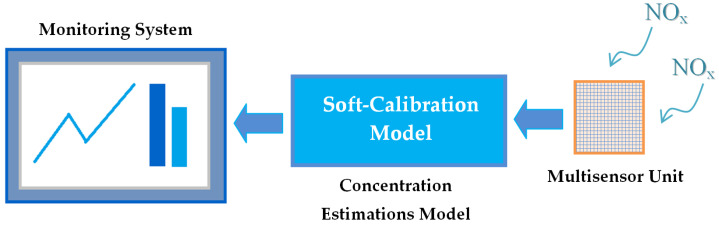
Block diagram of an electronic nose system that combines a soft-calibration model and a multisensor unit.

Engineering application of the EFO-GS optimization was demonstrated for the training of a PWM neuron to obtain the soft-calibration model for improvement of NO_x_ measurements by using a low-cost multisensor array. Figure 17 depicts a block diagram of the electronic nose that combines a soft-calibration model and a multisensor unit. The experimental study on the air quality dataset revealed that the PWM neuron model with EFO-GS optimization can improve the accuracy of NO_x_ measurements from solid state sensor arrays, and it can be implemented as an integral part of electronic nose applications. This study illustrated the performance of this soft-calibration model to estimate NO_x_ concentration measurements in the range of [14, 368] ppb from multisensor array data. However, the PWM neuron with EFO-GS can be used to generate a soft-calibration model for other gases (CO, NO_2_, NMHC) so that the reference chemical analyzer measurements are available in the dataset.

## Figures and Tables

**Table 1:** Advantages and disadvantages of some fundamental metaheuristic optimization methods that were used for the training of ANNs.

Metaheuristic Methods	Advantages	Disadvantages
PSO	For the training of shallow neural networks, the PSO can present faster convergence than backpropagation algorithms [14] and perform global searching [15].	Although performing a global search, it is possible to converge to local minima. Inappropriate selection of hyper-parameters of PSO may produce relatively poor results [18].
GA	The GA can provide better training performance than backpropagation algorithms [13] because GA performs a gradient-free optimization [15] and global search. It can be effective for training of shallow neural networks.	The convergence to minimum solution can take longer when hyper-parameters are not well tuned [18].
DE	It can perform global searching in the training [23] and find optimal ANN training solutions at the expense of more computation time [27].	The DE algorithm may cause premature convergence and poor performance [18,28].

**Table 2:** Operation modes of power-weighted multiplicative neurons.

Intervals	Operation Modes	Interval Reversing to Switch between Operation Modes
x<0	Complex-valued Neuron	When x≥0 in the real valued mode, use −x as input data because −x<0
x≥0	Real-valued Neuron	When x<0 in the complex valued mode, use −x as input data because −x>0
$x_{j}<0$ and $x_{j}\geq 0$	Mixed-mode Neuron	It operates the mixed-mode when input data have positive and negative values

**Table 3:** Reduction of the PWM neuron model to other neuron models via the proper parameter setting.

Proper Parameter Configuration	Neural Network Model	Model Formulation
None	PWM neuron model	$v=\sum_{i=1}^{m}\left(w_{i}\prod_{j}^{h}x_{j}^{p_{i,j}}\right)+b$, $s=a_{1}z_{1\lambda }\left(v\right)+a_{1}z_{2\lambda }\left(v\right)$, y=φ(s)
h=1, $p_{i,j}=1$, $a_{1}=1$, $a_{2}=0$ and λ=c	McCulloch and Pitts classical neuron model	$v=\sum_{i=1}^{m}\left(w_{i}x_{i}\right)+b$, $s=v_{r}$, y=φ(s)
$p_{i,j}\in Z^{+}$, $a_{1}=1$, $a_{2}=0$ and λ=c	Polynomial neurons	$v=\sum_{i=1}^{m}\left(w_{i}\prod_{j}^{h}x_{j}^{p_{i,j}}\right)+b$, $s=v_{r}$, y=φ(s)

**Table 4:** Air quality dataset for the training of the PWM neuron model.

Sensor Types	Data Types	Explanation	Parameters
PT08.S1	Input	Tin oxide gas sensor (CO sensitive)	$x_{1}$
PT08.S2	Input	Titania gas sensor (NMHC sensitive)	$x_{2}$
PT08.S3	Input	Tungsten oxide gas sensor (NO_x_ sensitive)	$x_{3}$
PT08.S4	Input	Tungsten oxide gas sensor (NO_2_ sensitive)	$x_{4}$
PT08.S5	Input	Indium oxide gas sensor (O_3_ sensitive)	$x_{5}$
Temperature	Input	Temperature Measurement °C	$x_{6}$
Relative Humidity	Input	Relative Humidity Measurement (%)	$x_{7}$
Absolute Humidity	Input	Absolute Humidity	$x_{8}$
Reference Analyzer	Ground truth data	True concentration measurements for NO_x_ (ppb)	$y_{d}$

**Table 5:** Mean Square Error (MSE), Mean Absolute Error (MAE), Mean Relative Error (MRE), and R^2^ score performances of the tested models.

Estimation Models	MSE	MAE	MRE	R^2^
ANN	1079.7	22.8	0.15	0.84
GP	1038.9	24.6	0.18	0.84
PWM-EFO	725.52	20.8	0.16	0.89

**Table 6:** Mean and standard deviations of measurement errors for three models.

Estimation Models	Mean	Standard Deviation
ANN	11.9	30.6
GP	−6.9	31.5
PWM-EFO	2.75	26.8

**Table 7:** Mean Square Error (MSE), Mean Absolute Error (MAE), Mean Relative Error (MRE), and R^2^ score performances of tested models.

Estimation Models	MSE	MAE	MRE	R^2^
PWM-EFO (Real)	725.52	20.8	0.16	0.89
PWM-EFO (Complex)	842.46	22.9	0.18	0.87

## Data Availability

Not applicable.

## Appendix A

**Remark** **A1.**
*On the basis of the Lyapunov stability theorem, a condition for evolutionary energy minimization in the field can be expressed as*

$$\begin{array}{c} \Delta F_{k}\left[n\right]=F\left(X_{k}\left[n+1\right]\right)-F\left(X_{k}\left[n\right]\right)<0. \end{array}$$

**Proof.** The negative derivative of evolutionary energy function minimizes the evolutionary energy. Let us take a Lyapunov energy function as the evolutionary energy $F(X_{k}\left(t\right))>0$. For convergence to the minimum, the energy function should satisfy the stability criterion

$$\begin{array}{c} \frac{dF(X_{k}\left(t\right))}{dx}<0. \end{array}$$

One can substitute the finite difference for discretization of the derivative operator as follows

$$\begin{array}{c} \frac{dF(X_{k}\left(t\right))}{dx}=\lim_{h\to 0}\frac{F\left(X_{k}\left[n+h\right]\right))-F\left(X_{k}\left[n\right]\right))}{h}<0. \end{array}$$

For a sequential unit time increment, *h* can be set to 1 in order to represent discrete evolution seasons.

$$\begin{array}{c} \frac{dF(X_{k}\left(t\right))}{dx}≃F\left(X_{k}\left[n+1\right]\right))-F\left(X_{k}\left[n\right]\right))<0 \end{array}$$

Accordingly, the following condition minimizes evolutionary energy at each discrete evolution season:

$$\begin{array}{c} \Delta F_{k}\left[n\right]=F\left(X_{k}\left[n+1\right]\right)-F\left(X_{k}\left[n\right]\right)<0 \end{array}$$

□

**Remark** **A2.**
*A geometrical crossover of all agent property codes $X_{i}\left[n\right]$ towards the seasonal best agent code $X_{best}\left[n\right]$, at a scale of the magnitude of quality factor $\left|Q[n]\right|$, maximizes the total quality factor (($\sum_{i=1}^{h_{k}}Q_{i}\left[n\right]$) at each seasonal evolution. The maximum total quality factor in seasonal evolution is*

$$\begin{array}{c} max\sum_{i=1}^{h_{k}}Q_{i}\left[n\right]=\sum_{i=1}^{h_{k}}\frac{\left|F\left(X_{best}\left[n\right]\right)-F\left(X_{k}\left[n\right]\right)\right|}{\left|F(X_{best}\left[n\right])\right|+\left|F(X_{k}\left[n\right])\right|}. \end{array}$$

**Proof.** Let us assume that property codes of $X_{i}\left[n\right]$ change toward $X_{best}\left[n\right]=\underset{X_{j}}{argmin}F\left(X_{j}\left[n\right]\right)$ at the season *n*. The seasonal evolutionary quality factor is written according to Equation (7)

$$\begin{array}{c} Q_{i}\left[n\right]=-\frac{F\left(X_{best}\left[n\right]\right)-F\left(X_{k}\left[n\right]\right)}{\left|F(X_{best}\left[n\right])\right|+\left|F(X_{k}\left[n\right])\right|}. \end{array}$$

Since the selection mechanism guarantees the selection of advantageous evolution that satisfies the condition $\Delta F_{i}=F_{i}\left(X_{best}\right)-F_{i}\left(X_{i}\right)<0$ in the algorithm, one can easily write the quality factor as

$$\begin{array}{c} Q_{i}\left[n\right]=-\frac{-\left|F\left(X_{best}\left[n\right]\right)-F\left(X_{k}\left[n\right]\right)\right|}{\left|F(X_{best}\left[n\right])\right|+\left|F(X_{k}\left[n\right])\right|}=\frac{\left|F\left(X_{best}\left[n\right]\right)-F\left(X_{k}\left[n\right]\right)\right|}{\left|F(X_{best}\left[n\right])\right|+\left|F(X_{k}\left[n\right])\right|}. \end{array}$$

Hence, for the selection of advantageous evolution, one can write $\sum_{i=1}^{h_{k}}Q_{i}\left[n\right]=\sum_{i=1}^{h_{k}}\left|Q_{i}\left[n\right]\right|$. In addition, it is apparent that $\left|F\left(X_{best}\left[n\right]\right)-F\left(X_{i}\left[n\right]\right))\right|\geq \left|F\left(X_{i}\left[n\right]\right)-F\left(X_{k}\left[n\right]\right)\right|$, because of $X_{best}\left[n\right]=\underset{X_{j}}{argmin}F\left(X_{j}\left[n\right]\right)$. Then, the maximum value of total quality factor is written for the evolution towards $X_{best}$ in form of

$$\begin{array}{c} max\sum_{i=1}^{h_{k}}Q_{i}\left[n\right]=\sum_{i=1}^{h_{k}}\frac{\left|F\left(X_{best}\left[n\right]\right)-F\left(X_{k}\left[n\right]\right)\right|}{\left|F(X_{best}\left[n\right])\right|+\left|F(X_{k}\left[n\right])\right|}. \end{array}$$

Consequently, the seasonal evolution of all property codes towards $X_{best}\left[n\right]$ maximizes the total quality factor in the evolution process. □

**Remark** **A3.**
*The multiplicative unit, which is expressed as $u=\prod_{j=1}^{h}x^{p_{j}}$, turns into a weighted geometric average operator when the condition $\sum_{j=1}^{h}p_{j}=1$ is satisfied.*

**Proof.** One can write geometric average of parameters $x_{1},x_{2},x_{3},\ldots ,x_{h}$ as

$$\begin{array}{c} G_{m}={\left(\prod_{j=1}^{h}x_{j}\right)}^{\frac{1}{h}}=\prod_{j=1}^{h}x_{j}^{\frac{1}{h}}. \end{array}$$

Let us apply exponent weights $\alpha_{i}>0$ for each parameter $x_{j}$ as $x_{j}^{\alpha_{i}}$. The order-weighted geometric average of $x_{1},x_{2},x_{3},..,x_{h}$ series is written in the form of

$$\begin{array}{c} G_{0}=\prod_{j=1}^{h}x_{j}^{\frac{\alpha_{i}}{h}}. \end{array}$$

where the exponents $p_{j}=\frac{\alpha_{i}}{h}$ is the power weight. The function $G_{0}$ expresses a weighted geometric average and when the power weight satisfies the condition $\sum_{j=1}^{h}p_{j}=1$, Equation (20) performs an order-weighted geometric average [75,76]. □

**Proposition** **A1.**
*Assuming a negative real number ($x\in R^{-}$) and a non-integer power $p_{r}\in R-\left\{Z\right\}$, one can state that ($x^{p_{r}}$) can be written as the complex-valued parameter*

$$\begin{array}{c} x^{p_{r}}={\left|x\right|}^{p_{r}}\left(cos\left(\pi p_{r}\right)+jsin\left(\pi p_{r}\right)\right). \end{array}$$

**Proof.** Since the parameter *x* is a negative real number (x<0 and x∈R), one can write ($x=\left(-1\right)\left|x\right|$) and, then,

$$\begin{array}{c} x^{p_{r}}={\left(\left(-1\right)\left|x\right|\right)}^{p_{r}}={\left(-1\right)}^{p_{r}}{\left|x\right|}^{p_{r}}={\left(\sqrt{-1}\right)}^{2p_{r}}{\left|x\right|}^{p_{r}}={\left(j\right)}^{2p_{r}}{\left|x\right|}^{p_{r}} \end{array}$$

The property $j^{2}=e^{j\pi }$ is used in above expression, the $x^{p_{r}}$ can be written as

$$\begin{array}{c} x^{p_{r}}=e^{j\pi p_{r}}{\left|x\right|}^{p_{r}}. \end{array}$$

When the property $e^{j\pi p_{r}}=cos\left(\pi p_{r}\right)+jsin\left(\pi p_{r}\right)$ is considered, one obtains Equation (23) as

$$\begin{array}{c} x^{p_{r}}={\left|x\right|}^{p_{r}}\left(cos\left(\pi p_{r}\right)+jsin\left(\pi p_{r}\right)\right). \end{array}$$

□

**Theorem** **A1.**
*(Complex-valued Neuron Mode): A PWM neuron model, which is defined by Equations (21) and (22), can perform a complex-valued neuron mode that can be expressed in the form of*

$$\begin{array}{c} v=b+\sum_{i=1}^{m}\left(w_{i}\left(\prod_{k=1}^{h}{\left|x_{k}\right|}^{p_{i,k}}cos\left(\pi \sum_{l=1}^{h}p_{i,l}\right)\right)\right)+j\sum_{i=1}^{m}\left(w_{i}\left(\prod_{k=1}^{h}{\left|x_{k}\right|}^{p_{i,k}}sin\left(\pi \sum_{l=1}^{h}p_{i,l}\right)\right)\right). \end{array}$$

**Proof.** By using Equation (23) in Equation (21), one can rewrite Equation (21) in general form as

$$\begin{array}{c} u_{i}=\prod_{k=1}^{h}{\left|x_{k}\right|}^{p_{i,k}}e^{j\pi p_{i,k}}={\left|x_{j}\right|}^{p_{i,1}}{\left|x_{j}\right|}^{p_{i,2}}\ldots {\left|x_{j}\right|}^{p_{i,h}}e^{j\pi \left(p_{i,1}+p_{i,2}+\ldots +p_{i,h}\right)}=\left(\prod_{k=1}^{h}{\left|x_{k}\right|}^{p_{i,k}}\right)e^{j\pi \sum_{l=1}^{h}p_{i,l}}. \end{array}$$

Then, by using $e^{j\pi \sum_{l=1}^{h}p_{i,l}}=cos\left(\pi \sum_{l=1}^{h}p_{i,l}\right)+jsin\left(\pi \sum_{l=1}^{h}p_{i,l}\right)$, this equation can be expressed as

$$\begin{array}{c} u_{i}=\prod_{k=1}^{h}{\left|x_{k}\right|}^{p_{i,k}}\left(cos\left(\pi \sum_{l=1}^{h}p_{i,l}\right)+jsin\left(\pi \sum_{l=1}^{h}p_{i,l}\right)\right). \end{array}$$

When this equation is used in Equation (22), one obtains the output of the weighted sum in the form of

$$\begin{array}{c} v=b+\sum_{i=1}^{m}\left(w_{i}\left(\prod_{k=1}^{h}{\left|x_{k}\right|}^{p_{i,k}}cos\left(\pi \sum_{l=1}^{h}p_{i,l}\right)\right)\right)+j\sum_{i=1}^{m}\left(w_{i}\left(\prod_{k=1}^{h}{\left|x_{k}\right|}^{p_{i,k}}sin\left(\pi \sum_{l=1}^{h}p_{i,l}\right)\right)\right). \end{array}$$

□
